# Supplementary material for: Contractile ring mechanosensation and its anillin-dependent tuning during early embryogenesis
Source: Nat Commun. 2023 Dec 8;14:8138. doi: 10.1038/s41467-023-43996-4 (PMC10709429; doi:10.1038/s41467-023-43996-4)
Supplement: Supplementary file 1 — Supplementary information [file 41467_2023_43996_MOESM1_ESM.pdf]

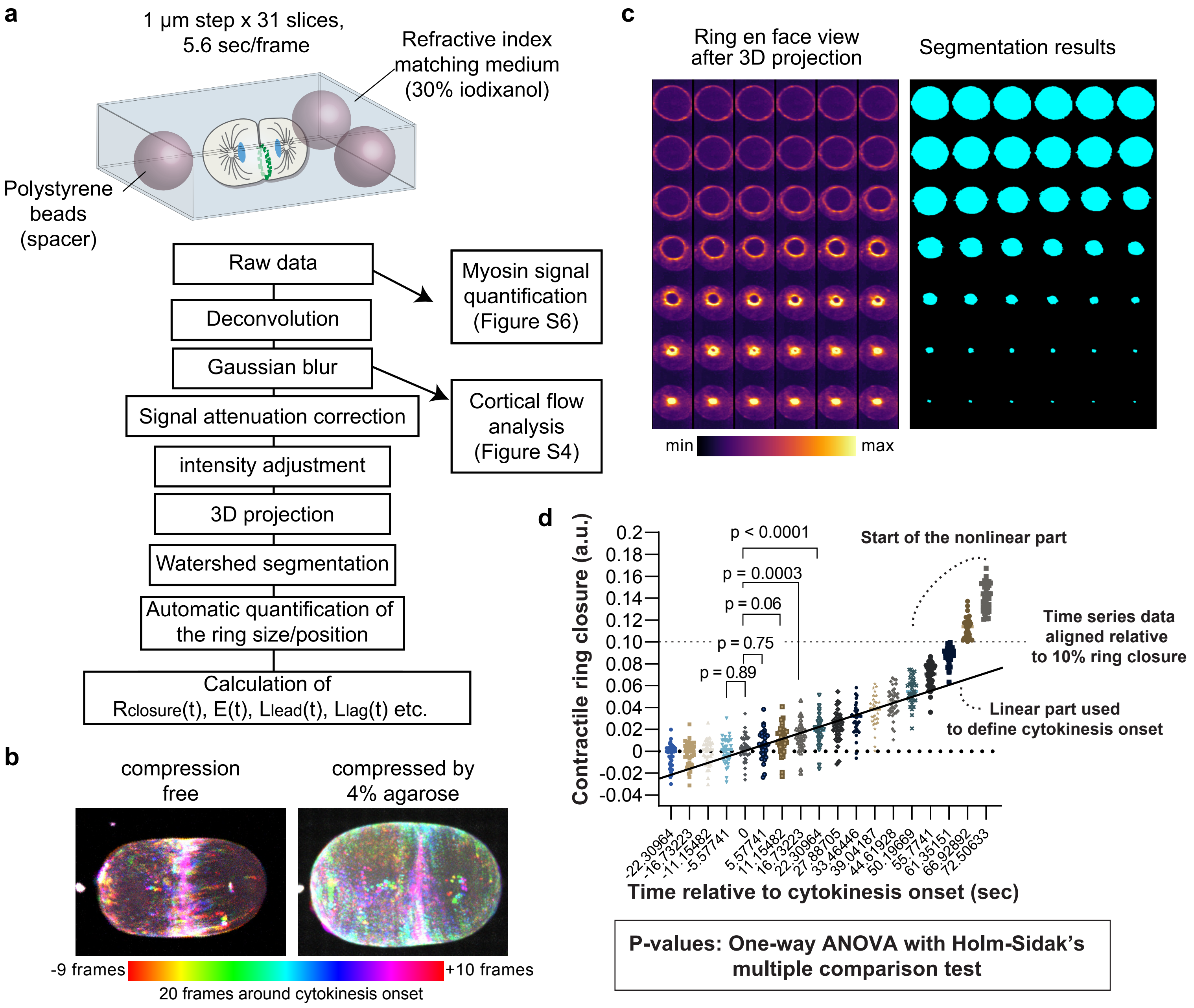

Supplementary Fig 1. Imaging and contractile ring analysis methods of P_0 zygotes.
(**a**) Imaging conditions and analytical flow charts. Refractive index matching medium and polystyrene beads were used to prevent light scattering and cellular compression, respectively. The use of a refractive index matching medium reduced the required slice number from 34 to 31 and also improved image resolution, especially on the deeper side of the cell (focal planes far from the objective lens). (**b**) Temporal color-coded image of 20 frames around cytokinesis onset, showing that compression-free embryos do not exhibit global cortical rotation. (**c**) Ring en face view images of 3D projected data and segmentation results. The first and last frames are the top left and bottom right in these figures, respectively. (**d**) Contractile ring closure curve after alignment of time series data relative to 10 % ring closure. Error bars indicate 95% confidence intervals. Cortical flow data and myosin signal intensity data were also aligned relative to 10% ring closure.

a

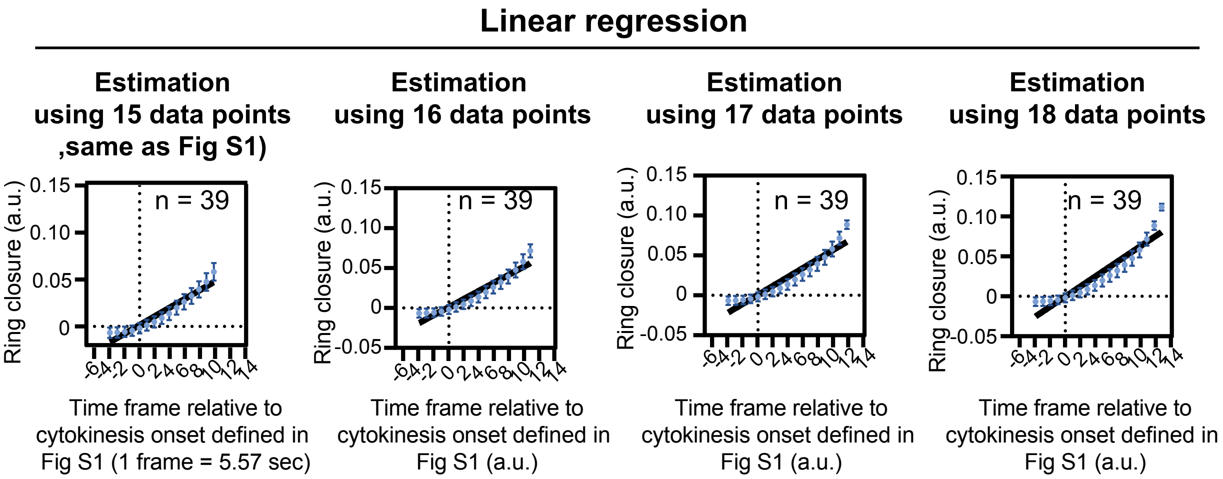

b

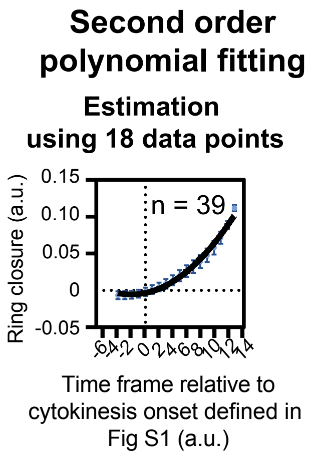

c

	15 data points	16 data points	17 data points	18 data points
Linear regression: Estimated x-intercept	-0.32 frame	-0.23 frame	-0.11 frame	+0.05 frame
Deviation from the cytokinesis onset defined in Fig. S1	N.A.	±0 frame	±0 frame	±0 frame
Second order polynomial fitting: Estimated x-intercept	+0.64 frame	+0.76 frame	+0.98 frame	+1.34 frame
Deviation from the cytokinesis onset defined in Fig. S1	+1 frame	+1 frame	+1 frame	+1 frame

Supplementary Fig. 2. Robustness of cytokinesis onset estimation method.
(**a**) Effects of parameter selection on the estimation of cytokinesis onset using simple linear regression. In this study, a linear segment of the ring closure curve was used for linear regression, as described in Figure S1. We subjectively determined the linear segment based on its shape, which included 15 data points (left-most graph). The subjective selection may affect the accurate estimation of cytokinesis onset. However, the selection of different numbers of data points had minimal effects on the estimated timing of cytokinesis onset (the x-intercepts of linear regression line). The x-axis represents the frame number of the time series data. (**b**) Estimation of cytokinesis onset using second order polynomial fitting. The black solid lines represent the fitted curves. (**c**) Summary of the effects of parameter selection on the of estimation of cytokinesis onset. Note that although the estimated x-intercepts (putative true cytokinesis onset) include decimals, the actual frame number in our time series data is an integer. Thus, when we obtained a -0.32 frame as the x-intercept, we defined the nearest integer frame, 0 frame, as the cytokinesis onset.

a

C. elegans gene name	Molecular identity of encoded proteins
ect-2	Ect2 RhoGEF
nop-1	Worm specific RhoA activator NOP-1
rga-3/rga-4	RhoGAP
pod-1	Coronin
arx-2	Arp2
let-502	ROCK
cyk-1	Formin
ani-1	Anillin
*unc-59**	Septin
nmy-2	non-muscle myosin II
mlc-4	myosin regulatory light chain
*act-2**	actin

***Redundantly working genes (unc-61/septin and actins act-1, act-3, act-4, act-5) were not analyzed.**

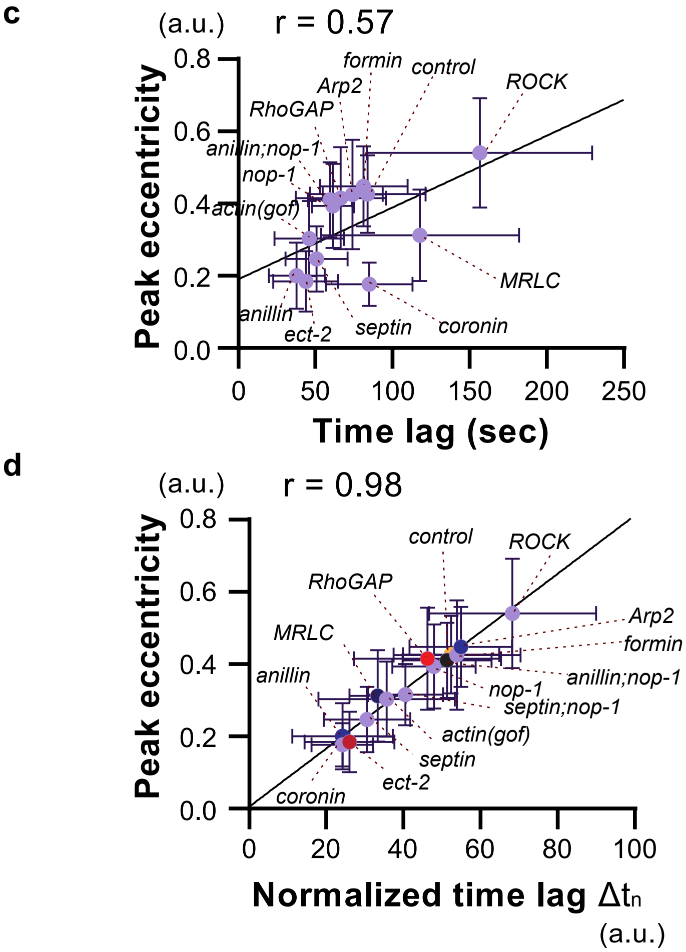

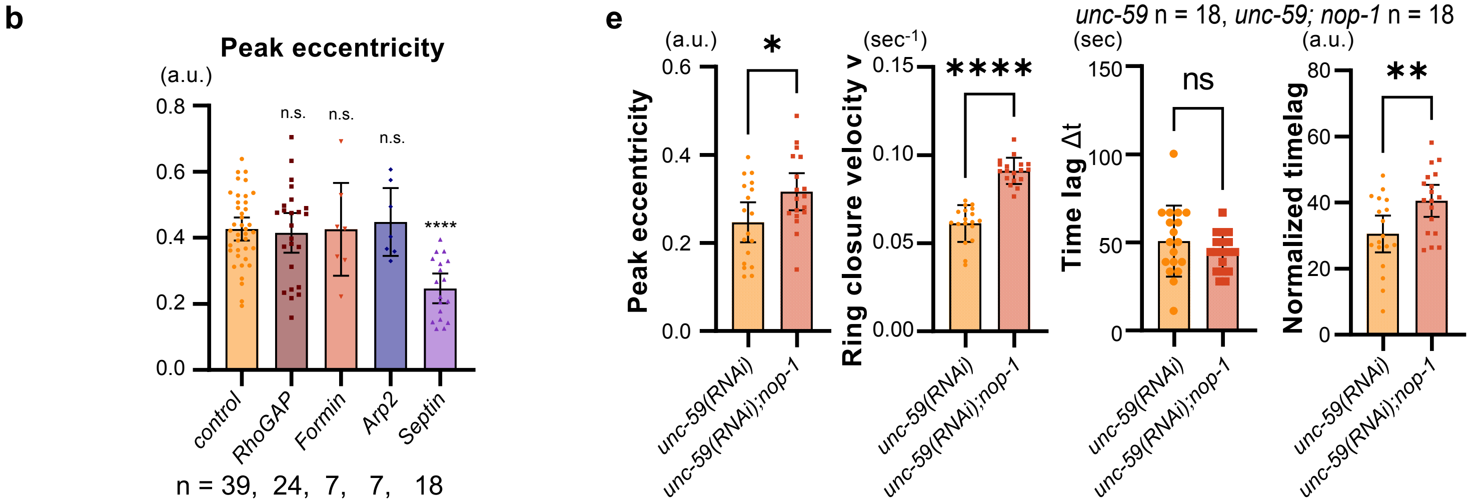

Supplementary Fig. 3. Additional genes analyzed to dissect contractile ring closure dynamics.
(**a**) A list of C. elegans genes analyzed in this study. (**b**) Peak eccentricity in different backgrounds. The control data is identical to what was used in Figure 2. (**c**) Relationship between peak eccentricity and time lag (raw values) in different backgrounds. (**d**) Relationship between peak eccentricity and normalized time lag in different backgrounds. The graph is essentially similar to Figure 2E but includes data from all the genes analyzed. (**e**) Rescue of unc-59/septin phenotype by nop-1 mutation. Similar to anillin, the peak eccentricity of unc-59 was rescued by the nop-1 mutation. Unlike the case of anillin, the rescue is likely to be due to the increase in ring closure velocity rather than the increase in time lag. In both anillin and septin, normalized time lag increased due to the nop-1 mutation. Times are relative to cytokinesis onset. p-values were calculated by one-way ANOVA with Holm-Sidak's multiple comparison test.

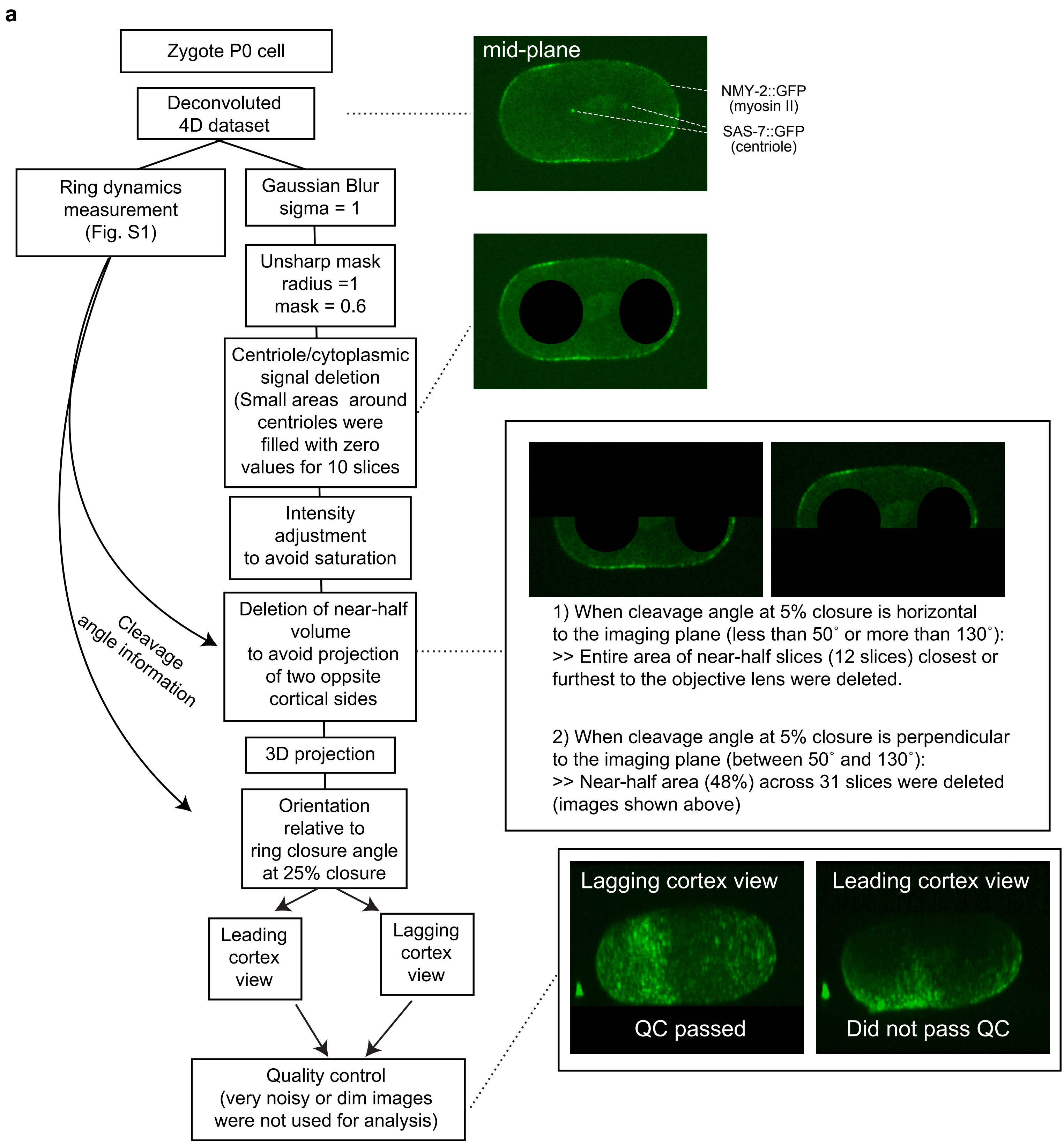

Supplementary Fig. 4. Generation of cortical myosin data used for PIV analysis.
(**a**) An image preprocessing pipeline used to generate cortical myosin data for Particle Image Velocimetry analyses. See also Methods section. At the quality control step, very noisy or dim images were not selected for PIV analyses. These images are usually from image planes furthest from the objective lens.

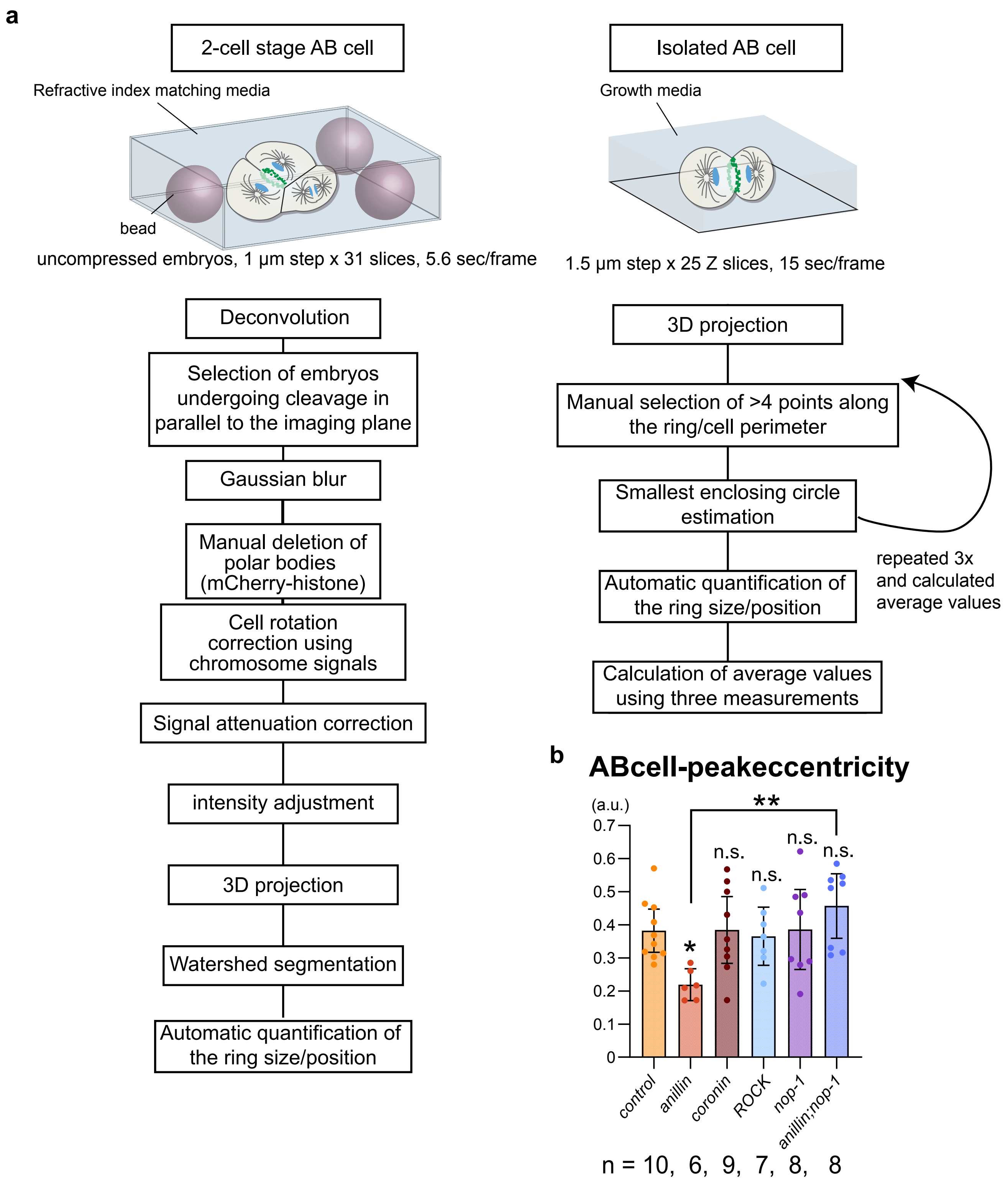

Supplementary Fig. 5. Imaging and contractile ring analysis methods of the two-cell stage AB cell. (**a**) Imaging conditions and analytical flow charts. Intact AB cells were analyzed similar to the P0 cells with some modifications to correct for cellular rotation. For isolated AB blastomeres, we used normal growth medium and a different analytical pipeline to estimate ring size and position due to the movement and rotation of the cells. Isolated AB cells were measured three times, and average values were analyzed. (**b**) Peak eccentricity of intact AB cells in different backgrounds. P-values were calculated by one-way ANOVA with Holm-Sidak's multiple comparison test.

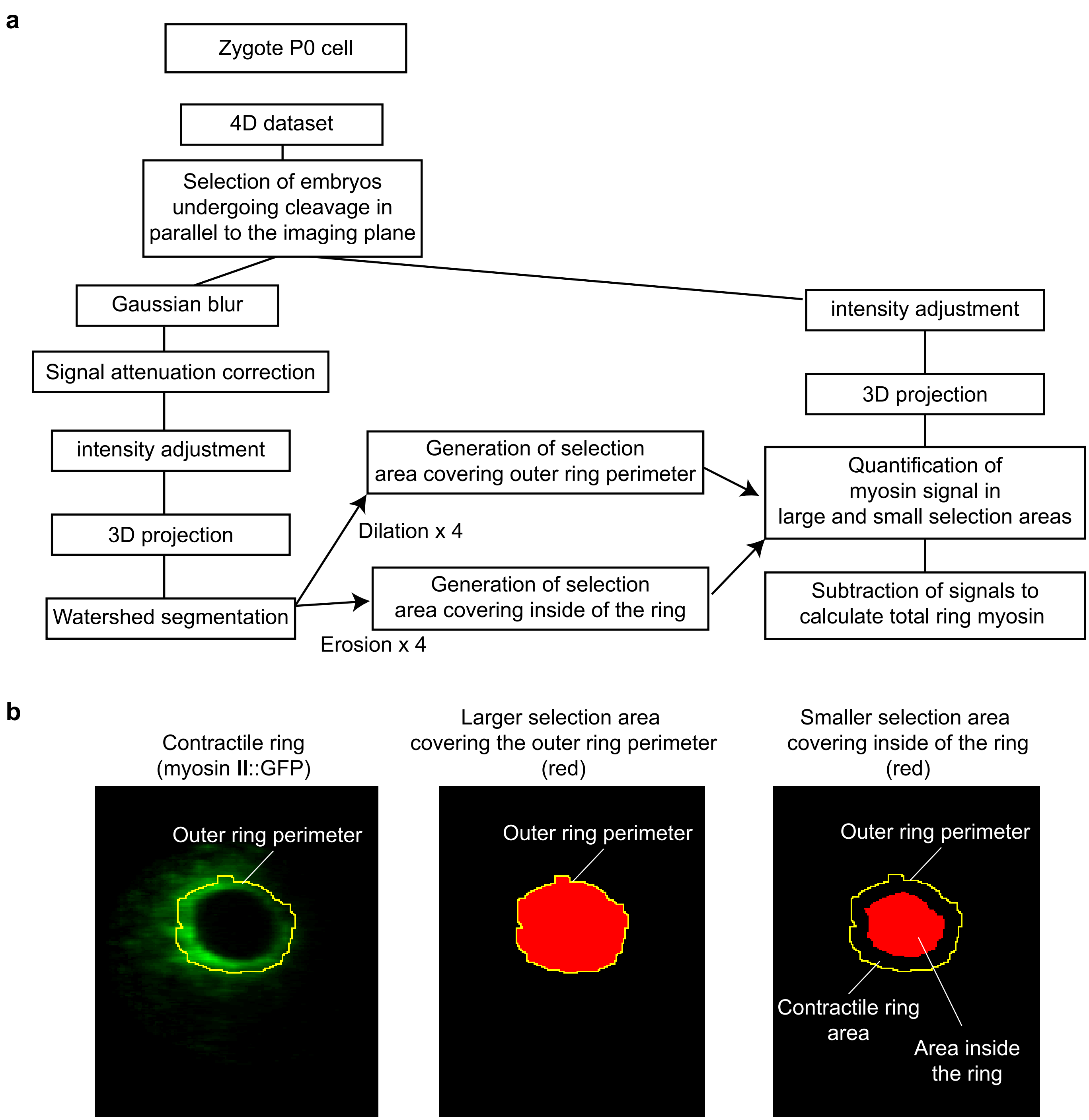

Supplementary Fig. 6. Quantification of total myosin signal in the ring.
(**a**) Image analysis pipeline for quantification of total myosin signal in the ring. We used the same 4D datasets as those used in the ring closure analysis and cortical flow analysis. Original 4D datasets exhibit attenuation effects, where image planes closer to the objective lens have higher signal compared to deeper planes. To mitigate this effect, we selected embryos undergoing cleavage parallel to the imaging plane for analysis. The segmentation method shown in Figure S1A was applied (left flow chart). The segmentation data were then dilated and eroded four times each to create selection areas that cover the outer ring perimeter and the inside of the ring. Myosin signal in these areas were measured using ring en face view images without Gaussian blur and attenuation correction. Total myosin signal in the ring was calculated by subtracting the value of the smaller selection area from the larger area. (**b**) Example of selection areas generated by the pipeline. Ring en face view generated after 3D projection (left). A larger selection area covering the outer perimeter of the contractile ring (middle; red). A smaller selection area covering the inside of the contractile ring (right; red).
