# Supplementary material for: Contractile ring mechanosensation and its anillin-dependent tuning during early embryogenesis
Source: Nat Commun. 2023 Dec 8;14:8138. doi: 10.1038/s41467-023-43996-4 (PMC10709429; doi:10.1038/s41467-023-43996-4)
Supplement: Supplementary file 3 — Description of Additional Supplementary Files [file 41467_2023_43996_MOESM3_ESM.pdf]

Description of Additional Supplementary Files

File Name: Supplementary Data 1
Description: Sequences for RNAi constructs

File Name: Supplementary Movie 1
Description: **Dynamics of cortical myosin and contractile ring of a single embryo**
Left top: maximum projection of cell surface non-muscle myosin II::GFP. Bottom left: ring en face view. Bottom middle: segmented ring. Bottom right: reconstructed ring trajectory. Right graph: Contractile ring dynamics of the same embryo. Note that the same embryo was used to generate all data.

File Name: Supplementary Movie 2
Description: **Cortical flow at the leading and lagging cell cortex**
3D projection of cell surface non-muscle myosin II::GFP. Times are relative to cytokinesis onset.

File Name: Supplementary Movie 3
Description: **Cortical flow and flow vectors estimated by Particle Image Velocimetry**
The cell surface myosin II::GFP of leading (left), lateral (middle), and lagging cortex views (right). Flow vectors (yellow arrows) are estimated by Particle Image Velocimetry. Times are relative to cytokinesis onset.

File Name: Supplementary Movie 4
Description: **Ring-directed cortical flow in MRLC knockdown**
3D oblique view and ring en face view of myosin II::GFP.

File Name: Supplementary Movie 5
Description: **Circumferential cortical flow in control RNAi embryos**
Surface of lagging equatorial cortex and 3D oblique view of myosin II::GFP.

File Name: Supplementary Movie 6
Description: **Dynamics of cortical compression and cortical ingression in *act-2(or295)***
Cell surface (top) and ring en face view (bottom left) of myosin II::GFP. Ring outlines and centroid trajectory were color-coded as in Figure 6E (yellow: upward movement, blue: bottomward movement).
