# Supplementary material for: Contractile ring mechanosensation and its anillin-dependent tuning during early embryogenesis
Source: Nat Commun. 2023 Dec 8;14:8138. doi: 10.1038/s41467-023-43996-4 (PMC10709429; doi:10.1038/s41467-023-43996-4)
Supplement: Supplementary file 11 — Reporting Summary [file 41467_2023_43996_MOESM11_ESM.pdf]

nature portfolio

Corresponding author(s): Kenji Sugioka

Last updated by author(s): Nov 16, 2023

Reporting Summary

Nature Portfolio wishes to improve the reproducibility of the work that we publish. This form provides structure for consistency and transparency in reporting. For further information on Nature Portfolio policies, see our Editorial Policies and the Editorial Policy Checklist.

Statistics

For all statistical analyses, confirm that the following items are present in the figure legend, table legend, main text, or Methods section.

n/a	Confirmed	
☐	☒	The exact sample size (*n*) for each experimental group/condition, given as a discrete number and unit of measurement
☐	☒	A statement on whether measurements were taken from distinct samples or whether the same sample was measured repeatedly
☐	☒	The statistical test(s) used AND whether they are one- or two-sided<br>*Only common tests should be described solely by name; describe more complex techniques in the Methods section.*
☒	☐	A description of all covariates tested
☐	☒	A description of any assumptions or corrections, such as tests of normality and adjustment for multiple comparisons
☐	☒	A full description of the statistical parameters including central tendency (e.g. means) or other basic estimates (e.g. regression coefficient) AND variation (e.g. standard deviation) or associated estimates of uncertainty (e.g. confidence intervals)
☐	☒	For null hypothesis testing, the test statistic (e.g. *F*, *t*, *r*) with confidence intervals, effect sizes, degrees of freedom and *P* value noted<br>*Give P values as exact values whenever suitable.*
☒	☐	For Bayesian analysis, information on the choice of priors and Markov chain Monte Carlo settings
☒	☐	For hierarchical and complex designs, identification of the appropriate level for tests and full reporting of outcomes
☐	☒	Estimates of effect sizes (e.g. Cohen's *d*, Pearson's *r*), indicating how they were calculated

Our web collection on statistics for biologists contains articles on many of the points above.

Software and code

Policy information about availability of computer code

Data collection	cellSense imaging (Olympus., inc) and Metamorph (Molecular Devices) were used for data collection as described in the Method section.
Data analysis	Fiji (open source), Prism 8 (Graphpad), Jupyter notebook (Project Jupyter), Matplotlib (ver 3.5.1), Scipy (ver 1.7.3), Numpy (ver 1.21.5), Pandas (ver 1.4.2) were used for data analysis as described in the Method section. Custom codes used in this paper is available: https://github.com/sugi01/unilateralcytokinesis

For manuscripts utilizing custom algorithms or software that are central to the research but not yet described in published literature, software must be made available to editors and reviewers. We strongly encourage code deposition in a community repository (e.g. GitHub). See the Nature Portfolio guidelines for submitting code & software for further information.

Data

Policy information about availability of data

All manuscripts must include a data availability statement. This statement should provide the following information, where applicable:

- Accession codes, unique identifiers, or web links for publicly available datasets
- A description of any restrictions on data availability
- For clinical datasets or third party data, please ensure that the statement adheres to our policy

Data is available as a source data file.

Research involving human participants, their data, or biological material

Policy information about studies with human participants or human data. See also policy information about sex, gender (identity/presentation), and sexual orientation and race, ethnicity and racism.

Reporting on sex and gender	N/A
Reporting on race, ethnicity, or other socially relevant groupings	N/A
Population characteristics	N/A
Recruitment	N/A
Ethics oversight	N/A

Note that full information on the approval of the study protocol must also be provided in the manuscript.

Field-specific reporting

Please select the one below that is the best fit for your research. If you are not sure, read the appropriate sections before making your selection.

☒ Life sciences ☐ Behavioural & social sciences ☐ Ecological, evolutionary & environmental sciences

For a reference copy of the document with all sections, see nature.com/documents/nr-reporting-summary-flat.pdf

Life sciences study design

All studies must disclose on these points even when the disclosure is negative.

Sample size	No statistical method was used to predetermine the sample size. We aimed to obtain around 10 samples for each treatment. For samples used to calculate cortical flow dynamics, the sample number is higher than others because we empirically know that these data are noisier than those of ring closure data.
Data exclusions	For PIV analysis, we excluded poor images that did not pass the quality control test (as described in Supplementary Fig. 4).
Replication	We have performed multiple independent experiments to collect data (ranging from 3-10 experiments to collect data of one genotype). Each experiment reproducibly showed the reported phenotypes.
Randomization	The experiments were not randomized.
Blinding	The investigators were not blinded to the study.

Reporting for specific materials, systems and methods

We require information from authors about some types of materials, experimental systems and methods used in many studies. Here, indicate whether each material, system or method listed is relevant to your study. If you are not sure if a list item applies to your research, read the appropriate section before selecting a response.

Materials & experimental systems

n/a	Involved in the study	
☒	☐	Antibodies
☒	☐	Eukaryotic cell lines
☒	☐	Palaeontology and archaeology
☐	☒	Animals and other organisms
☒	☐	Clinical data
☒	☐	Dual use research of concern
☒	☐	Plants

Methods

n/a	Involved in the study	
☒	☐	ChIP-seq
☒	☐	Flow cytometry
☒	☐	MRI-based neuroimaging

April 2023

Animals and other research organisms

Policy information about studies involving animals; ARRIVE guidelines recommended for reporting animal research, and Sex and Gender in Research

Laboratory animals	All C. elegans strains were cultured using the standard method 72. A temperature sensitive-actin mutant act-2(or295) was cultured at 15 ºC until the L4 larval stage and incubated at 25 ºC overnight before imaging. The following transgenes were used: cp13[nmy-2::GFP + LoxP] (non-muscle myosin II) 73, or1940[GFP::sas-7] (centriole marker) 74, itIs37 (mCherry::histone H2B), ruIs32[pie-1p::GFP::H2B::pie-1 3'UTR + unc-119(+)], knu83[cyk-1/formin::GFP]75, ca725[arx-2/Arp2::TagRFP]76, and ca973[GFP::pod-1/coronin]50. Other than itIs37, all the fluorescent reporters are endogenously tagged and were maintained as homozygotes. Ages of animals used for experiments: Zygote (fertilized egg) as stated in the manuscript.
Wild animals	N/A
Reporting on sex	Our research on cell division will use nematode C. elegans embryo as a model system. C. elegans has two different sexes—hermaphrodite and male. Hermaphrodites can generate both sperm and oocyte and self-fertilize. Our study will include both hermaphrodites and males, but because male populations are only 0.02%, 99.8% of our samples are hermaphrodites. There are methodological limitations in collecting only male embryos. However, timing and orientation of early embryonic divisions are almost identical between male and hermaphrodites, mainly due to the maternal contribution of protein and mRNA. Based on these reasons it is not applicable for us to integrate sex and gender differences in our cell division research, but our early embryonic division studies would not be affected by this.
Field-collected samples	N/A
Ethics oversight	N/A

Note that full information on the approval of the study protocol must also be provided in the manuscript.

Plants

Seed stocks	*Report on the source of all seed stocks or other plant material used. If applicable, state the seed stock centre and catalogue number. If plant specimens were collected from the field, describe the collection location, date and sampling procedures.*
Novel plant genotypes	*Describe the methods by which all novel plant genotypes were produced. This includes those generated by transgenic approaches, gene editing, chemical/radiation-based mutagenesis and hybridization. For transgenic lines, describe the transformation method, the number of independent lines analyzed and the generation upon which experiments were performed. For gene-edited lines, describe the editor used, the endogenous sequence targeted for editing, the targeting guide RNA sequence (if applicable) and how the editor was applied.*
Authentication	*Describe any authentication procedures for each seed stock used or novel genotype generated. Describe any experiments used to assess the effect of a mutation and, where applicable, how potential secondary effects (e.g. second site T-DNA insertions, mosiacism, off-target gene editing) were examined.*
